# Editorial: The clinical application and progress of precise diagnosis and treatment of thyroid tumors

**DOI:** 10.3389/fendo.2023.1214596

**Published:** 2023-05-17

**Authors:** Jiajia Tang, Jiaojiao Ma, Shirui Huo, Xuehua Xi, Huilin Li, Liangkai Wang, Zhe Sun, Xinyi Liu, Bo Zhang

**Affiliations:** ^1^ Department of Ultrasound, China-Japan Friendship Hospital, Beijing, China; ^2^ Chinese Academy of Medical Sciences and Peking Union Medical College, Beijing, China; ^3^ Beijing University of Chinese Medicine, Beijing, China; ^4^ Institute of Clinical Medicine, China-Japan Friendship Hospital, Beijing, China; ^5^ Capital Medical University, Beijing, China; ^6^ Department of Ultrasound, China-Japan Friendship Hospital, National Center for Respiratory Medicine, National Clinical Research Center for Respiratory Diseases, Institute of Respiratory Medicine of Chinese Academy of Medical Sciences, Beijing, China

**Keywords:** thyroid tumors, targeted imaging, gene analysis, etiology factors, prognostic model, minimally-invasive therapy

Thyroid tumors are common and have become a significant public health concern worldwide. Precise diagnosis and treatment of thyroid tumors are essential for effective disease management and improved outcomes. In recent years, significant advancements in medical research and technology have led to the development of new diagnostic and treatment strategies for thyroid tumors.

One of the most promising developments of thyroid tumors is the application of genetic testing and targeted diagnosis. This has enabled clinicians to identify high-risk patients and tailor treatments to the specific genetic mutations in the thyroid tumors. Ma et al. described the development of a targeted microbubble contrast agent for specific contrast-enhanced ultrasound (CEUS) imaging of highly invasive thyroid cancer. The microbubbles were prepared by coupling a peptide with high affinity and specificity for β-galactoside-binding protein galectin-3 (Gal-3) to the surface of lipid microbubbles and demonstrated good stability and biosafety. The microbubbles showed superior targeting ability to Gal-3 overexpressing BCPAP cells, significantly improving the CEUS imaging of highly invasive thyroid cancer in a mouse model. This study provides an innovative approach for the specific ultrasound imaging diagnosis of highly aggressive thyroid cancer, which assists in improving the precise diagnosis of thyroid tumors and reducing unnecessary treatment interventions. In a separate study, Yan et al. predicted the invasiveness of papillary thyroid microcarcinoma (PTMC) by combining ultrasound and the WNT10A gene analysis to evaluate the invasive capability of PTMC. The expression level of WNT10A was higher in the invasive group than in the non-invasive group. The risk factors for PTMC invasiveness included age, maximum nodule diameter, microcalcification, ultrasound-suspected lymph node metastasis, and WNT10A expression. The combination of ultrasound and WNT10A gene analysis is a potential method for evaluating the invasive capability of PTMC. It could have clinical value in diagnosing and treating thyroid tumors.

Recently, the environment, air, geology, and basic human body parameters have been suggested as potential risk factors for developing thyroid cancer. However, thyroid cancer’s exact pathogenesis is complex, multifactorial, and not fully understood. Huo et al. collected incidence data from 487 cancer registries and evaluated the impact of factors such as air pollution, green space, economic status, and health care level on thyroid cancer using geographical detector analysis. Economic status and health care level were found to have the most significant influence on thyroid cancer. The study also found gender differences in the impact of certain factors and observed spatial heterogeneity in the incidence of thyroid cancer. The findings provide important insights into the environmental and social determinants of thyroid cancer in China. Meanwhile, Ahmadi et al. evaluated whether body mass index (BMI) at the time of initial thyroid nodule evaluation could be correlated with thyroid cancer risk in a cohort of 1,259 consecutive patients. No significant association was found between BMI and the diagnosis of benign or malignant thyroid nodules, nor did BMI predict aggressive thyroid cancer. The study suggests that real-time measurement of BMI at the time of thyroid nodule evaluation does not contribute to thyroid cancer risk assessment.

B-cell primary thyroid malignant lymphoma (BC-PTML) is a rare and challenging-to-manage form of thyroid cancer originating from B cells. Jin et al. developed and validated a nomogram to predict cancer-specific survival (CSS) in patients diagnosed with BC-PTML. They used data from 1,152 patients in the SEER database and established a stepwise Cox regression model to construct a nomogram for predicting 5-, 10-, and 15-year CSS. The nomogram included radiotherapy, surgery, stage, chemotherapy, subtype, and age. The novel model had improved discriminatory ability and clinical benefits compared with the traditional Ann Arbor staging system and could assist clinicians in developing effective individualized treatment strategies for patients with BC-PTML.

With the development of minimally invasive treatment, ultrasound-guided ablation’s therapeutic efficacy and safety in PTMC always attract significant attention. Juan et al. evaluate the long-term efficacy and safety of ultrasound-guided percutaneous laser ablation (PLA) for treating elderly patients with PTMC. This single-centre study in China found that ultrasound-guided PLA was effective and safe, achieving complete ablation in all 38 patients with no significant complications. By the end of 42 months after ablation, all nodules disappeared, and the volume reduction rates demonstrated sustained efficacy. This study suggests that ultrasound-guided PLA is a potential alternative treatment for elderly patients with PTMC who are ineligible for surgery.

In summary, the clinical application and progress of precise diagnosis and treatment strategies have significantly improved the management of thyroid tumors. This collection of Frontiers in Endocrinology focuses on targeted imaging, gene analysis, etiology factors, prognostic model and minimally-invasive therapy. These studies and technologies have paved the way for more tailored and effective treatments, leading to better patient outcomes. As medical research evolves, we can expect further advancements in thyroid tumor diagnosis and treatment.

## Author contributions

JT wrote the original draft. JM and SH revised the manuscript. XX, HL, LW, ZS and XL checked the grammar. BZ approved the submitted version.

